# Enzyme-catalysed synthesis of pyridines from biomass-derived feedstocks

**DOI:** 10.1039/d6ob00839a

**Published:** 2026-06-09

**Authors:** Victoria Sodré, Goran M. M. Rashid, Boriana V. Yotzova, Timothy D. H. Bugg

**Affiliations:** a Department of Chemistry, University of Warwick Coventry CV4 7AL UK T.D.Bugg@warwick.ac.uk; b Dickinson College Carlisle Pennsylvania USA

## Abstract

Pyridines are found in many pharmaceuticals and agrochemicals, but are synthesised from fossil fuel conversion. 2,4- and 2,5-pyridinedicarboxylic acids have been reported as products from bioconversion of renewable lignin feedstocks using engineered strains of *Rhodococcus jostii* RHA1 (Z. Mycroft *et al.*, *Green Chem.*, 2015, **17**, 4974–4979), but previously it has been uncertain whether the formation of the pyridine ring was assisted by enzyme catalysis. The 4,5-extradiol ring fission product of protocatechuic acid, 4-carboxy-2-hydroxymuconate 6-semialdehyde (CHMS) shows structural similarity to α-ketoglutaric acid, the substrate for reductive amination by glutamate dehydrogenase (GDH). Testing of five glutamate dehydrogenase (GDH) isozymes from *R. jostii* RHA1 revealed that GDH5 catalyses NADH-dependent reductive amination of CHMS, and its cyclisation to form a dihydropyridine product. The dihydropyridine can be oxidised to 2,4-pyridinedicarboxylic acid using recombinant *P. fluorescens* dye-decolorizing peroxidase DyP1B, providing a route to substituted pyridines from a renewable feedstock.

## Abstract 2

*Rhodococcus jostii* glutamate dehydrogenase 5 (GDH5) assists in the cyclisation of pyridine-dicarboxylic acids derived from lignin.

## Graphical abstract

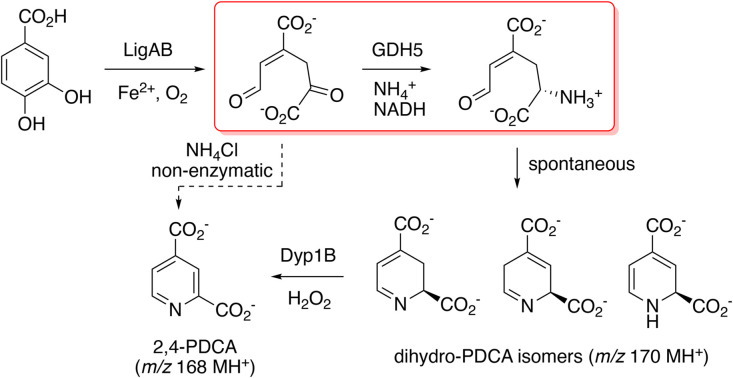

## Introduction

As modern society transitions towards a low-carbon economy to reduce greenhouse gas emissions, the chemical industry must find new, sustainable routes to high-value and feedstock chemicals that are currently manufactured from petrochemicals.^1,2^ Sustainable routes to aromatic chemicals from bio-based feedstocks are currently limited: there are routes to some bio-based substituted benzenes from aromatic amino acid biosynthesis;^3^ and routes to vanillin^4^ and substituted styrenes^5^ from degradation of lignin found in plant lignocellulose. However, for nitrogen-containing heterocycles such as pyridines and piperidines, which are found in many pharmaceuticals and agrochemicals, there is currently no sustainable route from a renewable source.^6^ Alkyl-substituted pyridines are prepared industrially by chemical oxidation of picolines or lutidines isolated from coal tar fractionation.^7,8^

**Fig. 1:**
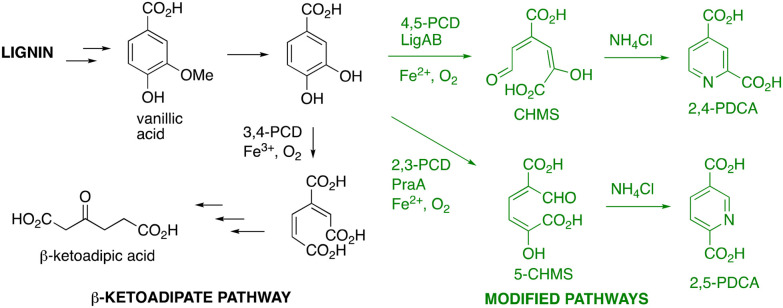
Pathway for formation of pyridine-dicarboxylic acid bioproducts from lignin by engineered *Rhodococcus jostii* RHA1. 4,5-PCD, proto-catechuate 4,5-dioxygenase; 2,3-PCD, protocatechuate 2,3-dioxygenase.

Our group has previously published the conversion of polymeric lignin or lignocellulose by engineered *Rhodococcus jostii* RHA1 into pyridine-dicarboxylic acid (PDCA) bioproducts.^9^ Instead of the normal 3,4-oxidative cleavage of key intermediate protocatechuic acid, insertion of genes encoding either protocatechuate 4,5-dioxygenase (encoded by *ligAB* genes from *Sphingobium* sp. SYK-6) or protocatechuate 2,3-dioxygenase (encoded by *praA* from *Paenibacillus* sp. JJ-1b) generates new extradiol oxidative cleavage products 4-carboxy-2-hydroxymuconate 6-semialdehyde (CHMS) and 5-carboxy-2-hydroxymuconate 6-semialdehyde (5-CHMS), which are cyclised in the presence of ammonium chloride present in M9 minimal media, to generate 2,4-pyridine-dicarboxylic acid (2,4-PDCA) or 2,5-pyridine-dicarboxylic acid (2,5-PDCA) respectively, as shown in Fig. 1.^9^ These compounds are analogues of terephthalic acid found in plastics such as PET and PBAT, and they can be converted using biocatalysis into PDCA-containing bioplastics.^10^ Titres of 60–125 mg L^−1^ were initially obtained by insertion of plasmid-borne *ligAB* or *praA* genes into wild-type *R. jostii* RHA1,^9^ which have been improved to 330 mg L^−1^ by gene deletion of the competing beta-ketoadipate pathway, and gene insertion onto the *R. jostii* chromosome.^11^

The molecular mechanism responsible for cyclisation of the pyridine ring during PDCA formation in *R. jostii* RHA1 was not certain.^9^ Although the non-enzymatic reaction of extradiol ring cleavage products with ammonium salts to form picolinic acids is precedented,^12,13^ it is slow at pH 7, and the same cyclisation occurs much more slowly in growing *Pseudomonas putida* cells,^14^ suggesting that the pyridine cyclisation reaction in *R. jostii* RHA1 is probably assisted in some way by enzyme catalysis. However, there are few precedents for enzyme-catalysed nitrogen heterocycle formation from acyclic substrates. For 6-membered nitrogen heterocycles, there is precedent for the use of ω-transaminase to convert a 1,5-dicarbonyl substrate into a 1,5-aminoketone, which then cyclises to a cyclic imine, and can then be reduced to a piperidine product.^15,16^ Cyclisation of a 1,2-diamine with a dicarbonyl compound to form a pyrazine heterocycle is also precedented,^17^ but there are no known examples of enzyme-catalysed cyclisation to form a pyridine product. For 5-membered nitrogen heterocycles, pyrrole formation is catalysed by transaminase enzyme PigE in the biosynthesis of a prodigiosin in *Serratia*,^18^ and by 5-aminolevulinic acid dehydratase in porphyrin biosynthesis,^19^ and formation of l-proline from l-ornithine is catalysed by ornithine cyclodeaminase.^20^ Here we report the identification of an enzyme responsible for pyridine ring formation from CHMS and 5-CHMS in *R. jostii* RHA1.

## Experimental section

### Bacterial strains and plasmids

The *ligAB* genes from *Sphingobium* sp. SYK-6 (1339 bp), encoding protocatechuate 4,5-dioxygenase LigAB, were cloned into pET-28b. The *praA* gene from *Paenibacillus* sp. JJ-1b (881 bp), encoding protocatechuate 2,3-dioxygenase PraA, was subcloned into pET28-a from pTipQC2 vector^9^ using restriction enzymes *Hind*III and *Nde*I, and confirmed by sequencing. The genes encoding GDHs 4–8 (GenBank accession numbers in Table 1) were codon-optimised for expression in *Escherichia coli*, synthesised, and cloned into the expression vector pET-151 by GeneArt (Life Technologies). *E. coli* BL21(DE3)pLysS (Invitrogen) cells were transformed with the respective plasmids by heat shock and plated on LBA media supplemented with 25 µg mL^−1^ chloramphenicol and 100 µg mL^−1^ ampicillin (for pET-151) or 50 µg mL^−1^ kanamycin (for pET-28a/b).

**Table 1:** Genes encoding glutamate dehydrogenase isozymes in *R. jostii* RHA1, their encoded proteins, and their expression in *E. coli* and activity for reaction with CHMS

Enzyme name	Gene ID	Amino acids	*M*_r_ (kDa)	Expressed?	Active with CHMS?
GDH1A	ro04644	1572	171	No	
GDH1B	ro03717	1542	168	No	
GDH1C	ro01009	1527	165	No	
GDH2	ro05607	1060	114	No	
GDH3	ro03471	1130	123	No	
GDH4	ro00339	382	39.5	Yes	No
GDH5	ro00573	429	45.5	Yes	Yes
GDH6	ro03288	122	12.9	Yes	Weakly
GDH7	ro08786	423	44.7	Yes	No
GDH8	ro01405	447	48.1	Yes	No

### Protein expression and purification

Cultures of the respective *E. coli* BL21-pLysS strains harbouring the desired expression plasmids were grown in lysogeny broth (also known as Luria–Bertani medium) (1 L) supplemented with 25 µg mL^−1^ chloramphenicol and either 100 µg mL^−1^ ampicillin or 50 µg mL^−1^ kanamycin, at 37 °C, 180 rpm. For LigAB and PraA expression, the media were also supplemented with 1 mM ammonium iron(ii) sulfate (final concentration). Protein expression was induced by addition of 0.5 mM isopropyl β-d-1-thiogalactopyranoside (IPTG) and left at 15 °C, 180 rpm, for 16–18 hours. Finally, the cultures were centrifuged, and the resulting cell pellets were either immediately used for purification, or frozen and kept at −20 °C until use.

For protein purification, cell pellets were resuspended in a final volume of 20 mL IMAC buffer (20 mM sodium phosphate buffer pH 7.4, 300 mM NaCl, 20 mM imidazole), supplemented with 1 mM phenylmethylsulfonyl fluoride (PMSF), 1 U mL^−1^ DNAse I, and 0.35 mg mL^−1^ lysozyme. For LigAB and PraA purification, the buffer was further supplemented with 1 mM 1,4-dithiothreitol (DTT). The resuspended cells were incubated for 30 min at room temperature, under agitation, and lysed using a E1061 cell disruptor (Constant Systems Ltd), at 20 kpsi. The lysed cells were centrifuged at 17 000 rpm, 4 °C, for 35 minutes. The supernatant containing soluble proteins was filtered through a 0.22 µm filter into a His GraviTrap™ column (Cytiva), pre-equilibrated with IMAC buffer. The column was washed with IMAC buffer containing increasing concentrations of imidazole: 20, 62.5, 125, 250, and 500 mM. The fractions from 250 and 500 mM washes were joined and concentrated using Amicon® Ultra-15 centrifugal filters, with 10 or 30 kDa cutoff, depending on the enzyme being purified. The concentrated fractions from IMAC purification were then buffer exchanged using PD-10 Sephadex™ G-25 columns (Cytiva) into 20 mM MOPS buffer pH 7.4 containing 150 mM NaCl and 15% glycerol, aliquoted, flash frozen and stored at −80 °C until use. The resulting purified proteins were analysed by SDS-PAGE and quantified by Bradford assay.

### Enzyme assays

Immediately prior to activity, 50 µL aliquots containing LigAB or PraA were thawed in ice and reactivated by incubating with 2.5 µL freshly prepared sodium ascorbate (100 mM), 2.5 µL freshly prepared ammonium iron(ii) sulfate (100 mM), on ice, for 15 minutes. The reconstituted enzymes were directly used for assays.

LigAB and PraA activities were assayed in 200 µL or 1 mL reactions containing 20 mM Tris-HCl buffer pH 8.0, 0.75–1 mM protocatechuic acid (PCA), and approximately 50 µg of the respective enzyme. The reactions were incubated at 30 °C for up to 30 min. The appearance of a bright yellow colour indicated dioxygenase activity, which was monitored by measuring the absorbance at 350 nm for 5-CHMS and 410 nm for CHMS.

Glutamate dehydrogenase activity was assayed using a Hidex Sense microtitre plate reader in 200 µL reactions containing 50 mM Tris-HCl pH 7.5, 1 mg ml^−1^ CHMS or 5-CHMS, 0.1 mM NADH (or NADPH), 20 mM NH_4_Cl, and 25–50 µg GDH5. Assays were incubated at 30 °C for up to 30 min and monitored by measuring the consumption of NADH at 340 nm. For determination of kinetic parameters, assays were carried out at a range of substrate concentrations: 0.625–10 mM for 5-CHMS and 0.03–1.0 mM for CHMS. The amount of NADH in µmoles was determined by fitting the data to the trendline equation derived from a NADH standard curve. Kinetic parameters (*K*_M_ and *k*_cat_) were determined by nonlinear regression fitted to the Michaelis–Menten equation using GraphPad Prism 8.

GABA transaminase activity was assayed in 200 µL reactions containing 50 mM Tris-HCl pH 7.5, 1 mg ml^−1^ CHMS or 5-CHMS, 1 mM GABA or l-glutamic acid, 50 µM pyridoxal 5′-phosphate, and 50 µg GABA-T enzyme. Reactions were monitored at 380 nm (at this wavelength there was less background absorbance due to PLP) over 30 min for reduction in absorbance of CHMS or 5-CHMS, and compared with control assays lacking enzyme.

### Large-scale dioxygenase reaction

For large-scale LigAB/PraA reaction, PCA (15 mg) was dissolved in 125 mL 20 mM Tris-HCl pH 8.0, and 0.5 mg reconstituted LigAB or PraA was added. The reaction was incubated at 30 °C for 30 minutes, giving a bright yellow solution. The reaction mixture was freeze-dried over 3 days to yield a yellow powder. This was resuspended in methanol (buffer salts and enzyme remained insoluble), and the solvent was removed *via* rotary evaporation under reduced pressure. The resulting material was analysed for the presence of PCA and CHMS/5-CHMS by ^1^H-NMR and LC-MS. The molar concentration of CHMS/5-CHMS was inferred based on the amount of residual PCA in the sample, which was quantified by HPLC analysis *via* comparison with a standard curve of authentic PCA.

### Larger-scale conversion by GDH5 and Dyp1B

5 mL reactions containing 90 µg GDH5, 5 mg CHMS or 5-CHMS, 0.1 mM NADH, and 20 mM NH_4_Cl were assembled in 50 mM Tris-HCl buffer pH 7.5 (in triplicate). Negative controls lacking GDH5 were also included. The reactions were incubated at 30 °C for 30 min and immediately extracted with isopropanol at high ionic strength as previously described.^11^ The resulting freeze-dried material was resuspended in methanol (to remove salts), filtered into a new vial, and the solvent removed by rotary evaporation. The resulting white solid containing GDH5 reaction products was resuspended directly in 100 mM sodium acetate buffer pH 5.5 for oxidation by Dyp1B.

*Pseudomonas fluorescens* Pf-5 Dyp1B was expressed and purified as previously described.^21^ A total of 0.5 mg and 1 mM H_2_O_2_ were added to the GDH5 reaction products resuspended in buffer, to a final volume of 1 mL. The reactions were incubated at 30 °C for 1 hour, freeze-dried, and the resulting solids resuspended in HPLC-grade methanol for HPLC and LC-MS analysis.

### Analytical methods

All high-performance liquid chromatography (HPLC) analyses were performed in a 1260 Infinity II LC system (Agilent). Hydrophilic Interaction Liquid Chromatography (HILIC) was performed using a SeQuant® ZIC®-cHILIC column, 3 µm, 100 Å, 250 × 4.6 mm (Supelco), with an isocratic elution of 80 : 20 (v/v) acetonitrile : ammonium acetate (5 mM) at a flow rate of 1 mL min^−1^, and a 5 µL sample injection volume. Detection was at 270 nm.

Reverse-phase high-performance liquid chromatography (RP-HPLC) was carried out using an Eclipse XDB-C18 column, 5 µm, 4.6 × 250 mm, (Agilent). The HPLC solvents were water + 0.1% formic acid (solvent A) and methanol + 0.1% formic acid (solvent B). The applied gradient was 5–100% B for 15 min, 100% B for 10 min, 100–5% B for 1 minute, and 5% B for 10 min, with a 1 mL min^−1^ flow rate and detection at 270 nm.

Liquid chromatography coupled to mass spectrometry (LC-MS) analyses were performed using a Bruker Amazon X ESI mass spectrometer, using the LC method given above. Dihydropyridines were identified by extracted ion analysis for *m*/*z* 170 (MH^+^), in positive ion mode. Pyridine-dicarboxylic acids were detected by extracted ion analysis for *m*/*z* 168 (MH^+^), in positive ion mode, and compared with authentic standards for 2,4- and 2,5-PDCA. NMR spectroscopy was performed on a Bruker Avance III HD 400 MHz instrument, in d_4_-methanol as solvent.

## Results

### Hypothesis for enzymes involved in nitrogen incorporation

**Fig. 2:**
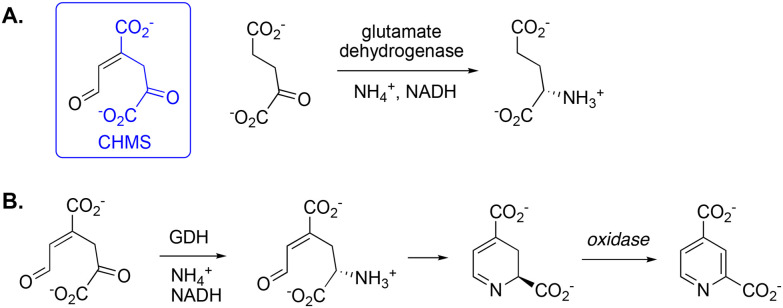
A. Structural similarity between the keto tautomer of extradiol ring fission product CHMS and α-ketoglutarate, the substrate for glutamate dehydrogenase. B. Hypothesis for conversion of CHMS by glutamate dehydrogenase (GDH) into dihydropyridine-2,4-dicarboxylic acid, then oxidation to 2,4-pyridinedicarboxylic acid.

An enzyme that is able to assist nitrogen incorporation from ammonium salts into ring fission product CHMS must be able to utilise either ammonium ions or perhaps pyridoxamine 5′-phosphate as substrate. It is known that incorporation of nitrogen in bacteria,^22,23^ yeast,^24^ and plants^25^ takes place primarily *via* biosynthesis of the amino acids glutamic acid and glutamine, *via* the enzymes glutamate dehydrogenase and glutamine synthetase. We noticed that there is a structural similarity between ring fission product CHMS and α-ketoglutarate, the substrate for glutamate dehydrogenase, as shown in Fig. 2A. Hence the incorporation of nitrogen by glutamate dehydrogenase in response to CHMS and ammonium chloride was a possible hypothesis for nitrogen incorporation, which would yield a reduced dihydropyridine product, which could then be oxidised intracellularly to form a pyridine product.

Analysis of the genome of *R. jostii* RHA1 revealed that this microbe contains 10 genes encoding putative glutamate dehydrogenase enzymes (see Table 1). Due to the high GC content of *Rhodococcus jostii* RHA1, protein expression of large proteins in *Escherichia coli* K12 is very challenging, so we designed constructs for the five GDH genes with enzyme *M*_r_ <100 kDa, for expression in *Escherichia coli*. Recombinant *R. jostii* RHA1 glutamate dehydrogenases 4–8 were successfully expressed as recombinant His_6_-tagged proteins, and purified by Ni^2+^ affinity chromatography (see SI Fig. S1).

### Testing of recombinant enzymes with extradiol ring fission products

In order to generate the ring fission product CHMS for enzyme assay, recombinant protocatechuate 4,5-dioxygenase (LigAB) was expressed in *E. coli* as a recombinant His_6_-tagged protein, and purified by Ni^2+^ affinity chromatography (see SI Fig. S2). The recombinant enzyme was found to be active after reactivation with 1 mM iron(ii) sulfate, whereupon reaction with protocatechuic acid in 20 mM Tris buffer pH 8.0 gave rise to the characteristic bright yellow extradiol ring fission product CHMS, with *λ*_max_ 412 nm as reported previously.^26^ Although the CHMS product was not extracted into organic solvent after acidification, it could be isolated by lyophilisation and resuspension in methanol. The ^1^H NMR spectrum of the CHMS product showed signals at *δ*_H_ 9.05, 7.39 and 6.58 ppm corresponding to the aldehyde and alkene protons (see SI Fig. S3).

The 2,3-extradiol cleavage product 5-CHMS was also generated by expression of recombinant PraA in *E. coli*, followed by Ni^2+^ affinity chromatography. Incubation of recombinant PraA with protocatechuic acid in 20 mM Tris pH 8.0 gave rise to the ring fission product 5-CHMS, absorbing at *λ*_max_ 350 nm as reported previously,^27^ which was isolated as described above.

**Fig. 3:**
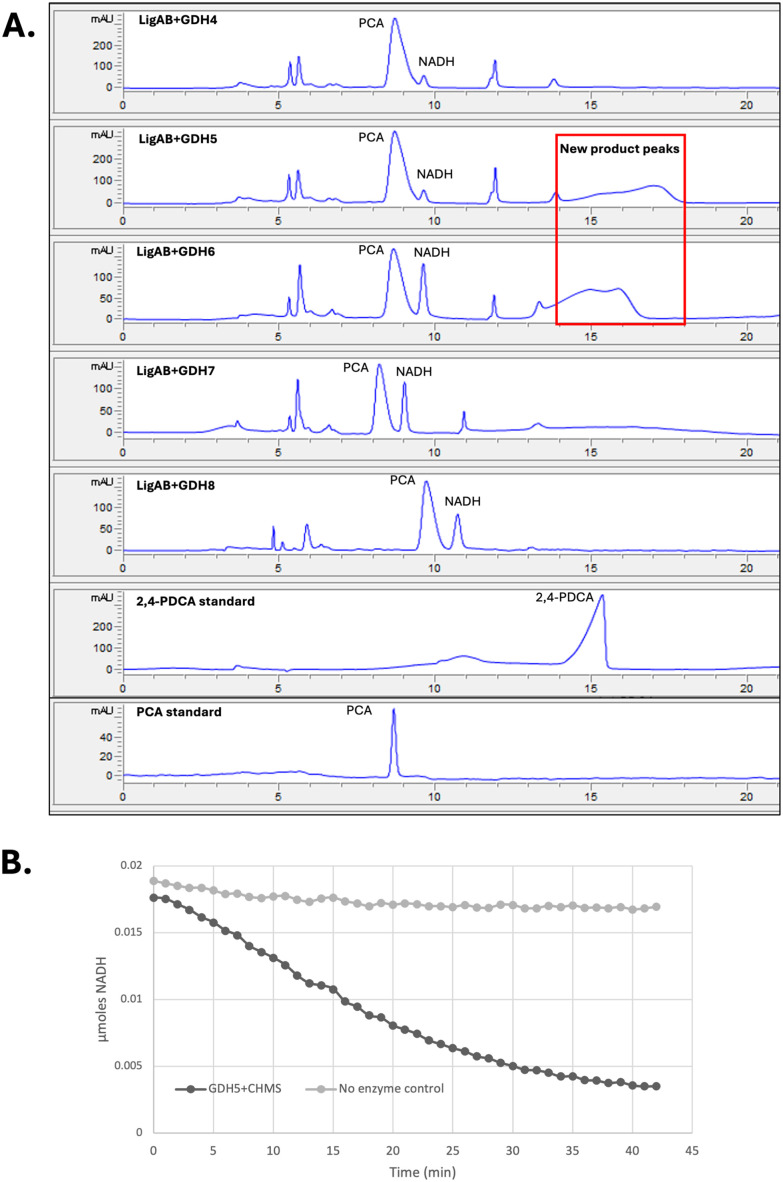
Testing of *R. jostii* GDH4–8 with CHMS as substrate. A, Observation of new product peaks by hydrophobic interaction liquid chromatography (HILIC). B, Enzyme-catalysed NADH consumption at 340 nm by reaction of 50 µg GDH5 with 1 mM CHMS.

Recombinant *R. jostii* RHA1 glutamate dehydrogenases 4–8 were screened for activity with CHMS, by incubation of each enzyme with PCA, LigAB, ammonium chloride, and 0.1 mM NADH. In the case of GDHs 5 and 6, the formation of new products with similar retention times to 2,4-PDCA was observed by hydrophobic interaction liquid chromatography (HILIC), accompanied by consumption of NADH (see Fig. 3A).

Recombinant *R. jostii* RHA1 GDH5 expressed well in *E. coli*, whereas GDH6 expressed poorly (see SI Fig. S1), so GDH5 was selected for further characterisation. As shown in Fig. 3B, time-dependent consumption of NADH was observed at 340 nm upon incubation of RjGDH5 with CHMS, with low background rate. Assaying GDH5 with either NADH or NADPH revealed that both cofactors could be utilised, with a preference for NADH, with highest activity at pH 7.5–8.0 (see SI Fig. S4).

The maximal rate of NADH consumption in the presence of CHMS by GDH5 was >80% of that observed for the natural substrate α-ketoglutarate (see SI Fig. S4). GDH5 also showed activity with 5-CHMS as substrate in the presence of NADH (see SI Fig. S4). GDH5 showed saturation kinetic behaviour with CHMS and 5-CHMS (see SI Fig. S5), and the steady-state kinetic parameters are shown in Table 2. CHMS shows lower estimated *K*_M_ than 5-CHMS, consistent with the closer structural resemblance to its natural substrate.

**Table 2:** Kinetic parameters for *R. jostii* RHA1 GDH5 using CHMS and 5-CHMS as substrates

Substrate	*K*_M_ (µM)	*k*_cat_ (min^−1^)
CHMS	45 ± 4	0.54 ± 0.03
5-CHMS	57 ± 5	0.38 ± 0.02
α-Ketoglutarate	8.2 ± 0.4	0.94 ± 0.04

**Fig. 4:**
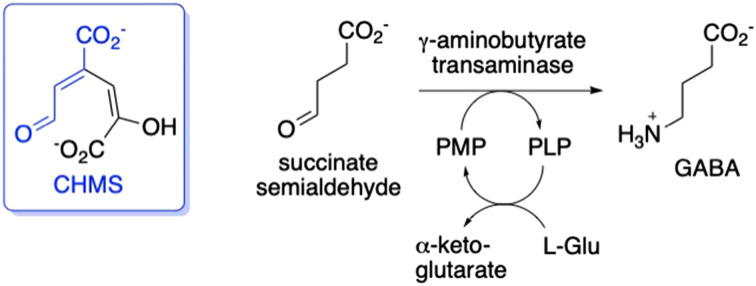
Possible structural similarity between the extradiol ring fission product CHMS and succinate semialdehyde, the substrate for γ-aminobutyrate (GABA) transaminase. PLP, pyridoxal 5′phosphate; PMP, pyridoxamine 5′-phosphate.

We also considered the possibility that enzyme-catalysed transamination might be taking place at the aldehyde terminus of CHMS. There is a possible structural resemblance of the aldehyde portion of CHMS to succinate semialdehyde, which is transaminated by γ-aminobutyric acid (GABA) transaminase to form GABA (see Fig. 4). There are four GABA transaminase genes in *R. jostii* RHA1 (ro04469, ro04544, ro05598, ro08787, GABA-T 1–4 respectively), whereas most bacteria contain only one GABA transaminase. Three *R. jostii* RHA1 GABA transaminases (GABA-T 1–3 as above) were successfully expressed as recombinant proteins in *E. coli*. Enzyme assay in the presence of CHMS or 5-CHMS with addition of GABA or l-glutamic acid showed consumption of substrate at 380 nm (see SI Fig. S6), but with a specific activity of only ∼0.2 nmoles per min per mg protein, compared with 10 nmoles per min per mg protein for GDH5. Therefore, although there is some observable activity of CHMS with GABA transaminase enzymes, it is much less than that observed for GDH5.

### Enzyme-catalysed formation of dihydropyridine and pyridine products

**Fig. 5:**
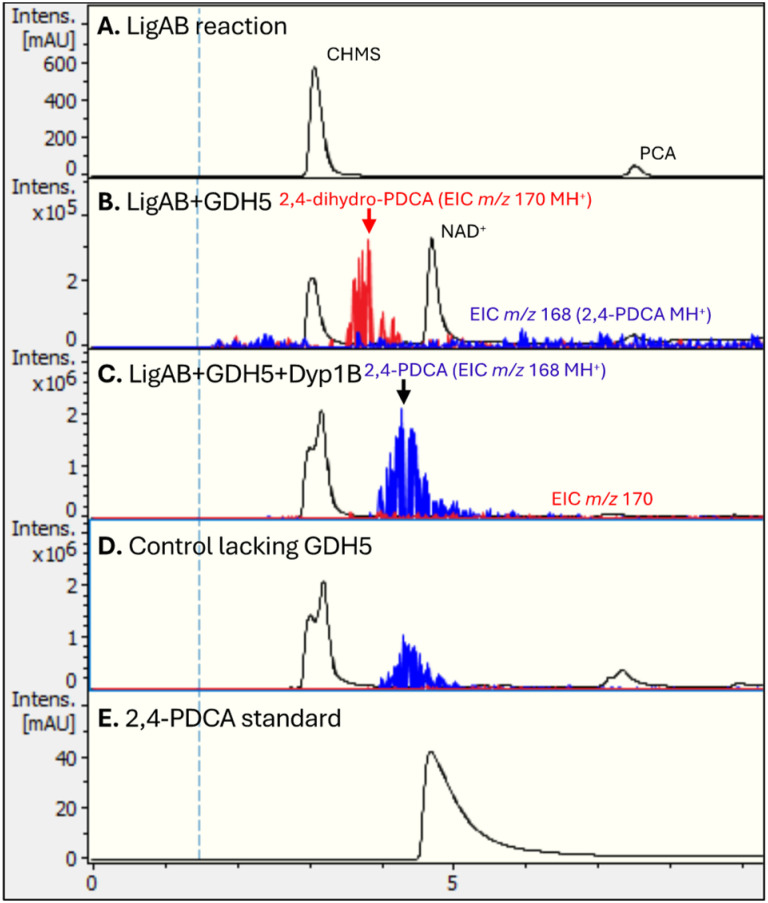
Generation of dihydropyridine and pyridine products by *R. jostii* RHA1 GDH5, observed by LC-MS. A, UV-vis trace (270 nm) for freeze-dried product of LigAB reaction, showing CHMS and remaining protocatechuic acid (PCA); B, reaction of CHMS with GDH5, showing the formation of dihydropyridine product, observed at *m*/*z* 170 (MH^+^); C, treatment of dihydropyridine product with *P. fluorescens* Dyp1B and hydrogen peroxide, showing formation of 2,4-pyridinedicarboxylic acid product at *m*/*z* 168 (MH^+^); D, control reaction lacking GDH5, showing the non-enzymatic formation of a smaller amount of 2,4-PDCA; E, authentic 2,4-pyridinedicarboxylic acid.

The formation of nitrogen heterocycles by GDH5 was then examined. Incubation of 1 mg mL^−1^ CHMS with *R. jostii* RHA1 GDH5 was found to give rise to a new peak at *m*/*z* 170 by LC-MS, corresponding to the MH^+^ form of a dihydropyridine product (see Fig. 5B). This product did not extract into organic solvents, consistent with existing as a zwitterion in aqueous solution, however we were able to extract into isopropanol at high ionic strength, a method that we have used previously for extraction of 2,4-PDCA,^11^ and then dissolve in methanol. The ^1^H NMR spectrum of this product showed new peaks at *δ*_H_ 7.7–7.8 ppm, consistent with an imine H̲C=N, but more than one set of peaks was observed, implying the existence of several isomers of the imine product (see SI Fig. S7).

Treatment of the dihydropyridine product with *P. fluorescens* peroxidase Dyp1B^21^ and hydrogen peroxide led to the formation of 2,4-pyridinedicarboxylic acid, observed at *m*/*z* 168 by LC-MS (see Fig. 5C). Co-injection of the GDH5/Dyp1B product with authentic 2,4-PDCA by HPLC verified their co-elution (see SI Fig. S8), confirming that the observed product is 2,4-PDCA. Analysis of the Dyp1B oxidation product by ^1^H NMR spectroscopy showed signals for the aromatic hydrogens of 2,4-PDCA at 8.61, 9.04 and 9.34 ppm (see SI Fig. S9).

We examined whether we could detect the non-enzymatic cyclisation of CHMS with ammonium chloride. No 2,4-PDCA was observed in the CHMS/GDH5 reaction after 30 min (see Fig. 5B, *m*/*z* 168), which suggested that the non-enzymatic cyclisation was slow. However, control reactions lacking GDH5 did gradually form a smaller peak for 2,4-PDCA (see Fig. 5D, *m*/*z* 168), to the extent of 25–30%, indicating the presence of a slower non-enzymatic reaction.

Reaction of GDH5 with 5-CHMS gave only weak signals at *m*/*z* 170 by LC-MS analysis (data not shown), consistent with 5-CHMS being a weaker substrate for GDH5, therefore there may be an alternative enzyme responsible for ammonia cyclisation of 5-CHMS to 2,5-PDCA in *R. jostii* RHA1.

**Fig. 6:**
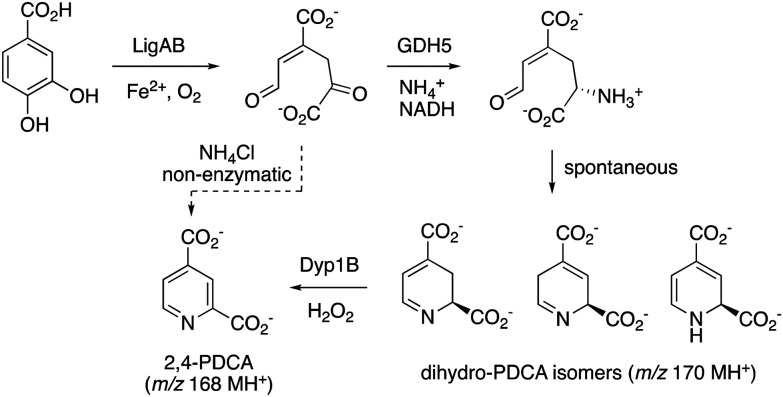
Proposed pathway for conversion of protocatechuic acid (PCA) into 2,4-pyridinedicarboxylic acid (2,4-PDCA) *via* RjGDH5 and Dyp1B. The non-enzymatic cyclisation of CHMS into 2,4-PDCA is also shown.

Therefore, we conclude that GDH5 uses CHMS as substrate for reductive amination, resulting in several dihydropyridine isomers (*m*/*z* 170 MH^+^). These reaction products can be oxidised by Dyp1B, generating 2,4-PDCA (*m*/*z* 168 MH^+^). A summary of this proposed pathway is shown in Fig. 6.

## Conclusions

In this manuscript we have identified an enzyme capable of catalysing the formation of a dihydropyridine ring from extradiol ring fission product CHMS, namely NADH-dependent glutamate dehydrogenase GDH5 in *R. jostii* RHA1. This enzyme may be responsible for the formation of 2,4-PDCA in engineered *R. jostii* RHA1 strains, from lignin feedstocks.^9,11^ The weaker cyclisation of PraA product 5-CHMS suggests that there may be a different enzyme responsible for cyclisation of 2,5-PDCA. There is no direct homologue for GDH5 in *P. putida* KT2440 (which contains two glutamate dehydrogenases: PP_0675, 449 aa; PP_2080, 1621 aa), which may explain the slow cyclisation of CHMS in the latter strain.^14^

We have shown that the dihydro-PDCA intermediate can be oxidised to the pyridine 2,4-PDCA by *P. fluorescens* Dyp1B.^21^*R. jostii* RHA1 contains two dye-decolorising peroxidases, DypA and DypB, which could catalyse this oxidation in *R. jostii* RHA1,^28^ but this strain also contains multi-copper oxidases McoA and McoC whose overexpression in *R. jostii* RHA1 has been shown to enhance titre of 2,4-PDCA,^29^ which might also catalyse this oxidation. The cyclisation of CHMS appears to generate more than one cyclic imine product, which could all be oxidised to form 2,4-PDCA.

Nevertheless, RjGDH5 activity could benefit from optimisation *via* protein engineering, not only to enhance activity, but to tailor its catalytic site to accept a wider range of substrates.

While ammonia cyclisation can happen non-enzymatically, several observations indicate that the non-enzymatic reaction is slower and less efficient than the GDH5-catalysed reaction: low background reaction in the GDH5 enzyme assay (Fig. 3B), and linear dependence of CHMS disappearance at 450 nm *vs*. GDH5 concentration (Fig. S4, panel F); no apparent formation of 2,4-PDCA in the presence of GDH5 but absence of Dyp1B (Fig. 5, panel B). But 25–30% formation of 2,4-PDCA was observed in the presence of Dyp1B but absence of GDH5 (Fig. 5, panel D), indicating that the background non-enzymatic reaction can be detected. Our conclusion is that the proportion of 2,4-PDCA product formed by non-enzymatic reaction in a microbial conversion will depend upon the amount of GDH5 enzyme present in the cell, or *in vitro* will depend upon the amount of GDH5 enzyme added.

Extradiol ring fission products are known to be prone to keto–enol tautomerisation,^30^ which was observed for CHMS and 5-CHMS (data not shown), and can show toxicity towards a microbial host.^14^ Thus, enzymes that can catalyse ammonia cyclisation of aromatic ring fission products such as RjGDH5 offer a more specific and efficient route to pyridines than non-enzymatic cyclisation, especially under physiological conditions – which is important for biotechnological applications.

A recent study has reported high-yield production of pyrone and pyridine dicarboxylic acids from glucose as feedstock in engineered *Corynebacterium glutamicum* strains.^31^ A BLAST search of RjGDH5 amino acid sequence against the *C. glutamicum* genome identified 17 matches with 25–32% sequence identity. By comparison, only 2 matches were identified when the same BLAST search was applied against the genome of *P. putida* KT2440. Considering that intense glutamate metabolism is a feature of *C. glutamicum*, as well as the high number of GDH isomers encoded in its genome, it is possible that some of these enzymes may have contributed towards pyridine cyclisation of the final products.

The identification of an enzyme capable of catalysing cyclisation of a pyridine ring opens up the possibility of generating a wider range of pyridines, from renewable sources, and hence could provide a new biocatalytic route to substituted pyridine chemicals.

## Author contributions

The primary laboratory-based research was carried out by V. S., G. M. M. R. and B. Y. Funding was obtained by T. D. H. B. The first draft of the manuscript was written by T. D. H. B. and V. S.

## Conflicts of interest

There are no conflicts to declare.

## Supplementary Material

OB-024-D6OB00839A-s001

## Data Availability

The primary data accompanying this article are shown in the supplementary information (SI). Supplementary information is available. See DOI: https://doi.org/10.1039/d6ob00839a.
