# Supplementary material for: Enzyme-catalysed synthesis of pyridines from biomass-derived feedstocks
Source: Org Biomol Chem. 2026 Jun 9;24(25):5326–32. doi: 10.1039/d6ob00839a (PMC13254512; doi:10.1039/d6ob00839a)
Supplement: OB-024-D6OB00839A-s001 [file OB-024-D6OB00839A-s001.pdf]

Supplementary Information (SI) for Organic & Biomolecular Chemistry.
This journal is © The Royal Society of Chemistry 2026

Enzyme-catalysed synthesis of pyridines from biomass-derived feedstocks

Victoria Sodré, Goran M.M. Rashid, Boriana Yotsova, and Timothy D.H. Bugg*

Supporting Information

Figure S1. Expression and purification of *R. jostii* RHA1 glutamate dehydrogenases GDH4-8.

Figure S2. Expression and purification of (A) *Sphingobium* sp. SYK-6 LigAB and (B) *Paenibacillus* sp. JJ-1b PraA.

Figure S3. Production of extradiol ring fission product CHMS by recombinant LigAB.

Figure S4. Assay of recombinant *R. jostii* RHA1 GDH5 with (A) α-ketoglutarate; (B) 4,5-ring fission product CHMS; (C) 2,3-ring fission product 5-CHMS.

Figure S5. Michaelis-Menten steady-state kinetic and Lineweaver-Burk double reciprocal plots for *R. jostii* RHA1 GDH5 with CHMS or 5-CHMS as substrates.

Figure S6. ^{1}H NMR spectrum of dihydropyridine product from reaction of CHMS with *R. jostii* GDH5.

Figure S7. Co-injection of GDH5/Dyp1B product with authentic 2,4-PDCA by C_{18} reverse phase HPLC.

	Name		bp	kDa
1	GDH4	ro0339	1149	39.5
2	GDH5	ro00573	1290	45.5
3	GDH6	ro03288	369	12.9
4	GDH7	ro08786	1272	44.7
5	GDH8	ro01405	1344	48.1

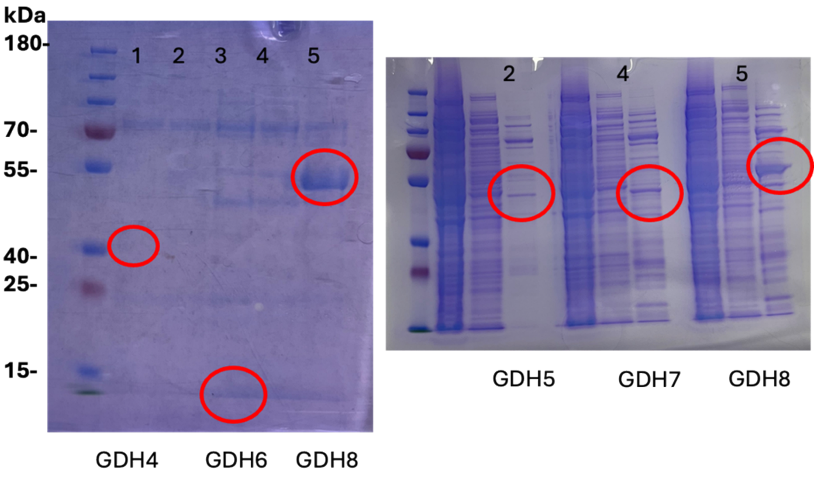

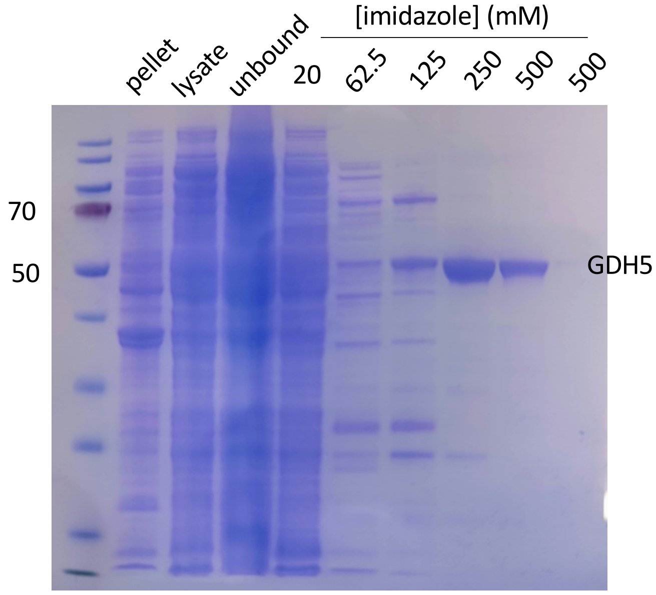

Figure S1. Expression and purification of *R. jostii* RHA1 glutamate dehydrogenases GDH4-8. Lower panel shows purification of *R. jostii* RHA1 GDH5 using gradient elution from Ni-NTA.

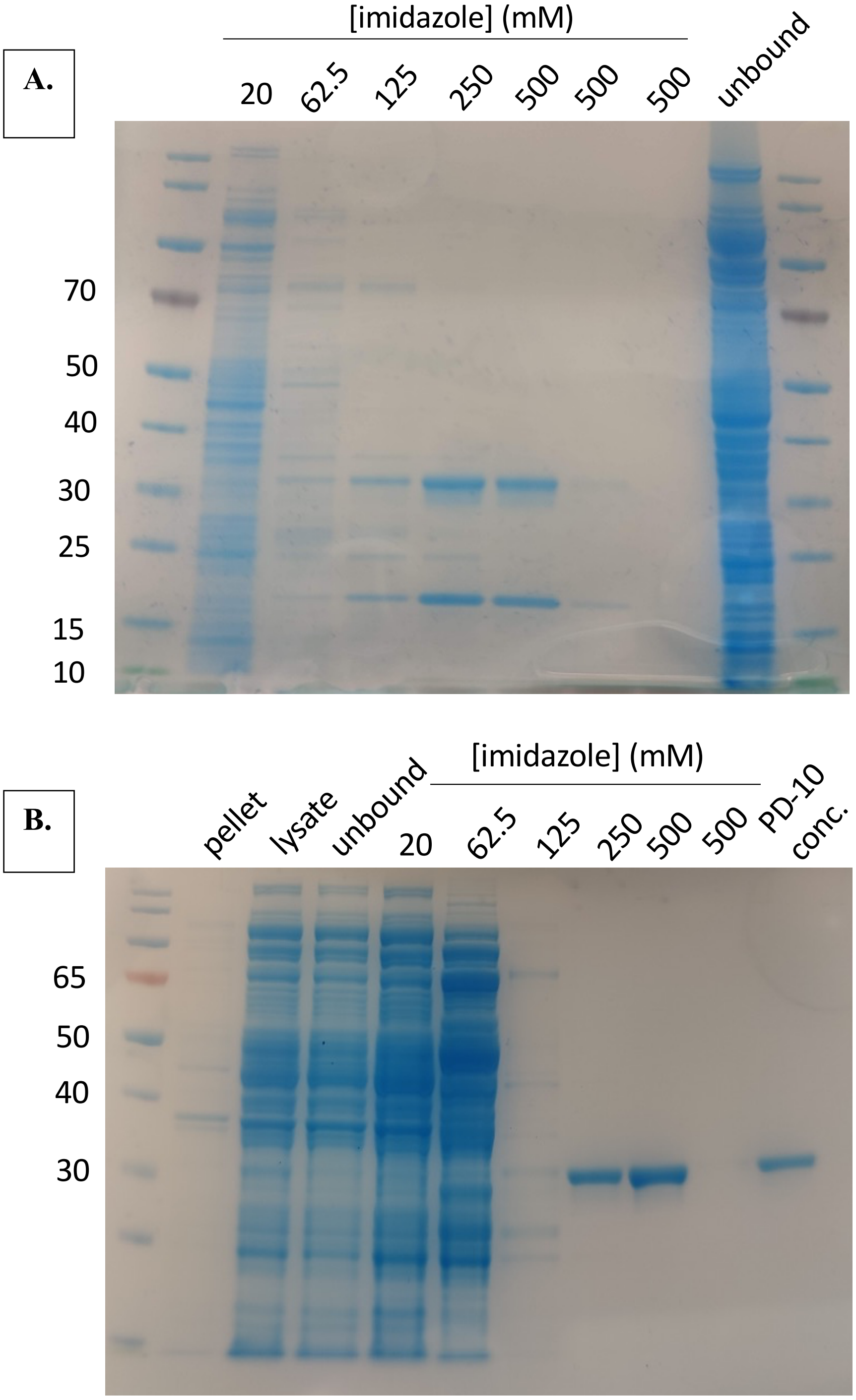

Figure S2. Expression and purification of (A) *Sphingobium* SYK-6 LigAB and (B) *Paenibacillus* PraA from *E. coli* BL21(DE3)/pLysS. LigA subunit = ~ 15 kDa, LigB subunit = ~30 kDa, PraA = ~30 kDa.

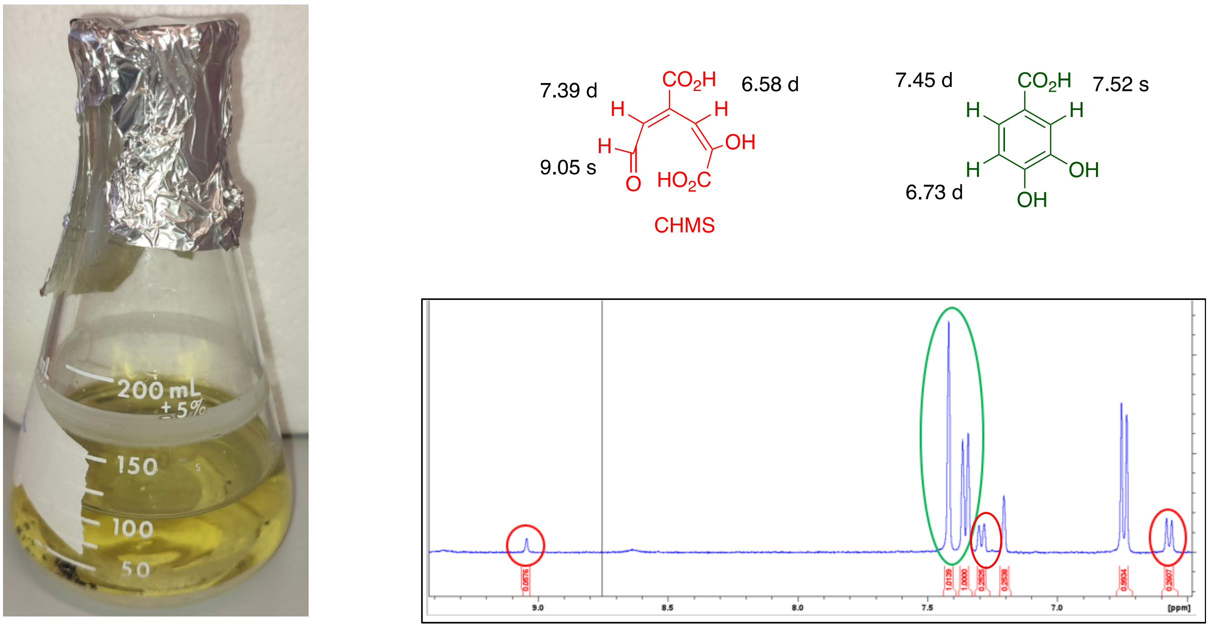

Figure S3. Large-scale (150 mL) production of extradiol ring fission product CHMS by recombinant LigAB (left), and ^{1}H NMR spectrum of CHMS (peaks highlighted in red, remaining protocatechuic acid in green.

Figure S4. Assay of 40 µg recombinant *R. jostii* RHA1 GDH5 with (A) 1.25 mM α-ketoglutarate; (B) 1 mM 4,5-ring fission product CHMS; (C) 1.25 mM 2,3-ring fission product 5-CHMS. (D) Cofactor preference determination using 5 mM α-ketoglutarate as substrate (NC = no enzyme control; statistical significance verified using Welch's t-test, * = p-value < 0.05, ** = p-value < 0.01). (E) pH profile using 5 mM α-ketoglutarate as substrate. The average and respective standard deviation of 3 replicates are shown. (F) Rate of consumption of CHMS (at 450 nm) vs. amount of GDH5 protein (in mg/ml).

Figure S5. Michaelis-Menten steady-state kinetic plots for *R. jostii* RHA1 GDH5 with CHMS or 5-CHMS as substrates.

A.

B.

Figure S6. Assay of *R. jostii* RHA1 GABA transaminase 1 (Panel A) or GABA transaminase 2 (Panel B) with 5-CHMS as substrate. Assays 1,2. GABA-T containing 1 mM GABA as nitrogen donor; control assay lacking enzyme. Assays 3,4. GABA-T containing 1 mM L-glutamic acid as nitrogen donor; control assay lacking enzyme. Assays contained 50 mM Tris-HCl pH 7.5, 1 mg/ml 5-CHMS, 1 mM GABA or L-glutamic acid, 50 µM pyridoxal 5'-phosphate, and 50 µg GABA-T enzyme, incubated for 30 min. Assays carried out in triplicate.

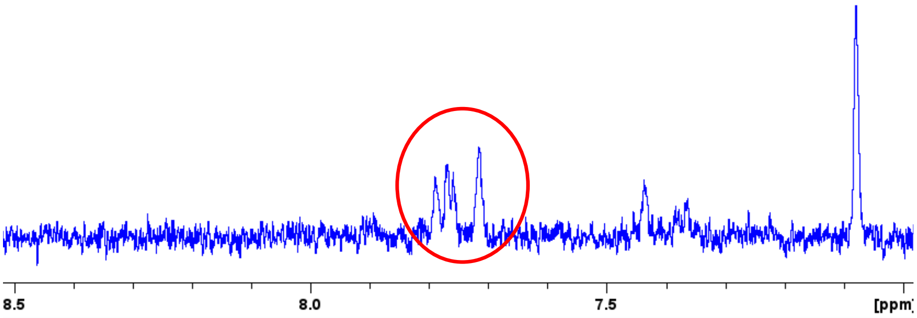

Figure S7. Aromatic region of ^{1}H NMR spectrum of dihydropyridine product from reaction of CHMS with *R. jostii* GDH5 (400 MHz, CD_3OD). Peaks at δ 7.7-7.8 ppm indicative of imine HC=N are highlighted.

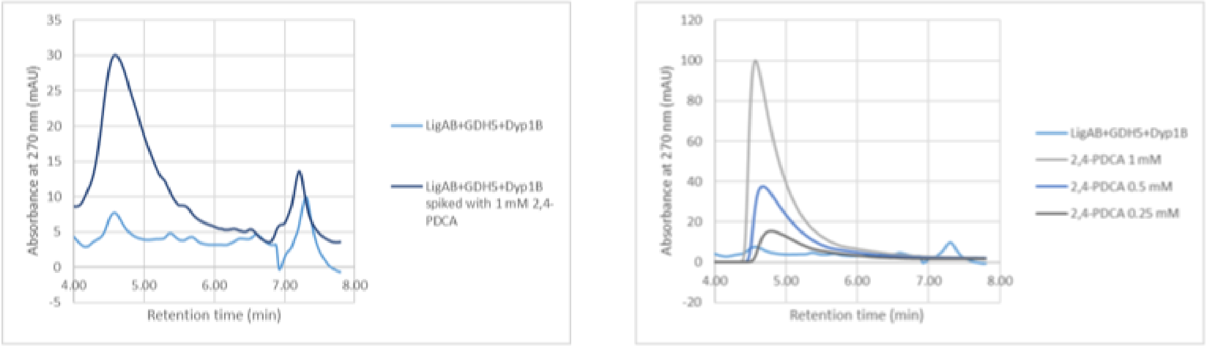

Figure S8. Co-injection of GDH5/Dyp1B product with authentic 2,4-PDCA by C_{18} reverse phase HPLC.

A. 2,4-PDCA generated from dihydro-DCA + Dyp1B (400 MHz, CD_3OD)

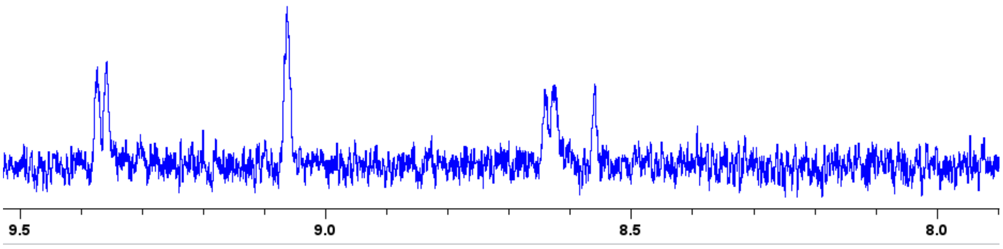

B. Authentic 2,4-PDCA (free acid, 400 MHz, CD_3OD)

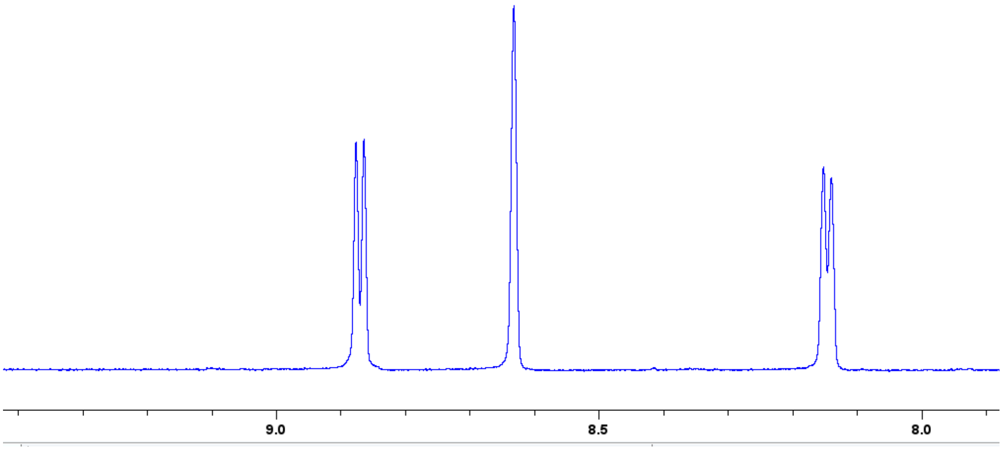

Figure S9. ^{1}H NMR spectra (400 MHz, CD_3OD) of (A) sample of 2,4-PDCA generated enzymatically from oxidation of dihydro-PDCA by *P. fluorescens* Dyp1B, extracted via isopropanol extraction from enzyme reaction mixture (present as the zwitterion) (B) authentic commercial sample of 2,4-PDCA (free acid).
